# 4D flow MRI of the aorta demonstrates age- and gender-related differences in aortic size and blood flow velocity in healthy subjects

**DOI:** 10.1186/1532-429X-17-S1-P39

**Published:** 2015-02-03

**Authors:** Julio Garcia, Kelly B Jarvis, Susanne Schnell, SC Malaisrie, Colleen Clennon, Jeremy D Collins, James C Carr, Michael Markl, Alex J Barker

**Affiliations:** 1 Radiology, Northwestern University, Chicago, IL, USA; 2 Biomedical Engineering, Northwestern University, Evanston, IL, USA; 3 Division of Cardiothoracic Surgery, Northwestern University, Chicago, IL, USA

## Background

Three-dimensional, time-resolved phase contrast MRI (4D flow) was applied to characterize the aortic size and 3D hemodynamics continuously along the centerline of the thoracic aorta. The aim was to investigate the impact of age and gender on the variability of normal aortic size and velocities.

## Methods

65 healthy subjects (age=43±14 yrs, 20 females) underwent aortic 4D flow MRI as part of an IRB-approved protocol. A 3D phase-contrast angiogram was generated from the 4D flow data followed by 3D segmentation of the aortic lumen. A volume centerline was calculated based on the aorta 3D segmentation and used to automatically characterize aortic diameter (AoD) and peak velocity (PV). Standardized anatomic landmarks (sino-tubular junction [STJ], branchiocephalic trunk [BCT], left subclavian artery [LSA], and descending aorta [DAo]) were used to normalize the measurements along the length of the aorta length. AoD and PV association with age and gender were assessed by Pearson's correlation and a Wilcoxon rank sum test was used to compare subjects by age (threshold 45 yrs) and gender.

## Results

**Figure 1:**
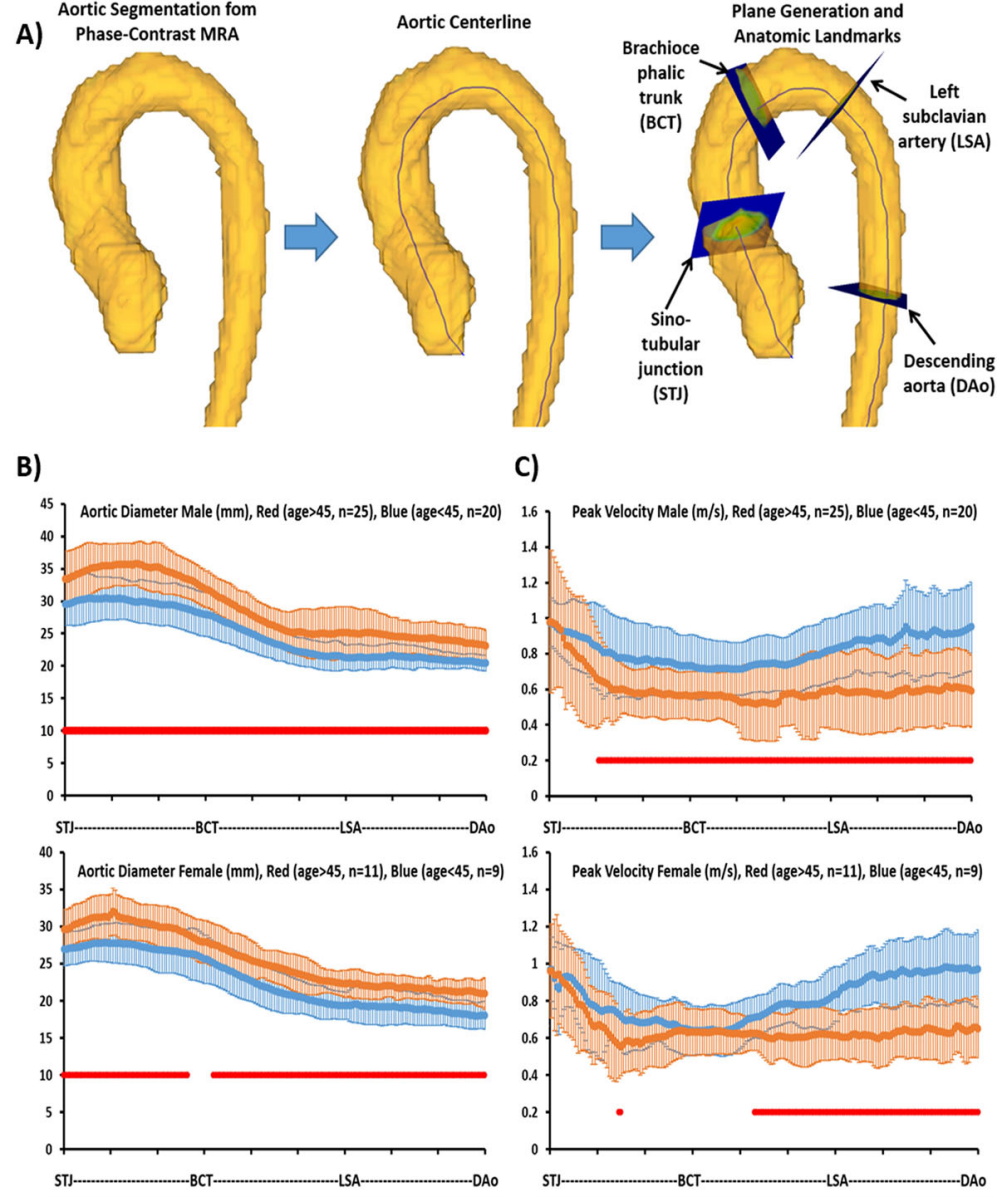
Automatic aortic diameter and peak velocity measurements. Phase-contrast MR angiogram (MRA) data from the 4D flow sequence was calculated and used to segment the entire aorta. Panel A shows example planes placed at landmark locations, and used along the entire centerline to measure aortic diameter and peak velocity. Diameter and velocity measurements are then computed from obtained planes and normalized to standard landmarks. Panel B shows the 95% confidence interval for the aortic diameter measurements by gender and age. Panel C shows the 95% confidence interval for the peak velocity measurements by gender and age. Red points indicates significant difference (p<0.05) between groups.

AoD and PV were successfully extracted along the entire thoracic aorta for all subjects (Fig. 1). AoD showed significant correlation with age (r=0.5, p<0.001), gender (r=0.43, p<0.001) and an inverse relationship with PV (r=-0.49, p<0.001). AoD for subjects under 45 yrs showed a better correlation (r=0.33, p=0.05) with age than those above 45 yrs (r=0.03, p=0.87). PV for subjects under 45 yrs showed an inverse relationship (r=-0.27, p=0.11) with age, similar to group above 45 yrs (r=-0.28, p=0.14). Significant differences were found between genders for AoD (p<0.001).

## Conclusions

Age and gender strongly influence regional differences of AoD and PV along the thoracic aorta. Establishing confidence intervals for these parameters, as a function of age and gender in healthy subjects is fundamental to the detection and classification of aortopathy in patient populations.

## Funding

This work was supported by NIH R01HL115828 and K25HL119608, AHA 13SDG14360004, AHA 14POST18350019, CONACyT (203355).

